# Chikungunya virus populations experience diversity- dependent attenuation and purifying intra-vector selection in Californian *Aedes aegypti* mosquitoes

**DOI:** 10.1371/journal.pntd.0007853

**Published:** 2019-11-21

**Authors:** Kasen K. Riemersma, Lark L. Coffey

**Affiliations:** Department of Pathology, Microbiology and Immunology, School of Veterinary Medicine, University of California, Davis, California, United States of America; Australian Red Cross Blood Service, AUSTRALIA

## Abstract

Chikungunya virus (*Togaviridae*, *Alphavirus*; CHIKV) is a mosquito-borne global health threat that has been transmitted transiently in the southeastern United States. A primary CHIKV mosquito vector, *Aedes aegypti*, was recently established in the populous state of California, but the vector competence of Californian mosquitoes is unknown. Explosive CHIKV epidemics since 2004 have been associated with the acquisition of mosquito-adaptive mutations that enhance transmission by *Ae*. *aegypti* or *Ae*. *albopictus*. As a highly mutable RNA virus, CHIKV has the potential for extensive and rapid genetic diversification in vertebrate hosts and mosquito vectors. We previously demonstrated that expansion of CHIKV diversity in cell culture allows for greater adaptability to novel selection pressures, and that CHIKV fidelity variants are able to diversify more than wildtype (WT) CHIKV in mice. The evolution of intra-vector CHIKV populations and the correlation between CHIKV population diversity and infectivity and transmissibility in mosquitoes has not yet been studied. Here, we address these gaps in knowledge via experimental infection of *Ae*. *aegypti* from California with WT and fidelity variant CHIKV. We show that *Ae*. *aegypti* from California are highly competent vectors for CHIKV. We also report that CHIKV fidelity variants diversify more than WT in mosquitoes and exhibit attenuated infectivity at the level of the midgut. Furthermore, we demonstrate that intra-vector populations of CHIKV are subjected to purifying selection in mosquito bodies, and sequences of non-coding CHIKV regions are highly conserved. These findings will inform public health risk assessment for CHIKV in California and improve our understanding of constraints to CHIKV evolution in mosquitoes.

## Author summary

Chikungunya virus (CHIKV) is transmitted by *Aedes aegypti* mosquitoes and has caused explosive epidemics in Asia and the Americas since 2004. During mosquito infection, the CHIKV genome replicates with a high mutation rate to produce virus populations with high genetic diversity that facilitate virus evolution. With this study, we address three gaps in knowledge: 1) are *Ae*. *aegypti* mosquitoes from Los Angeles, California, capable of transmitting CHIKV, 2) what effect does increased CHIKV population diversity have on virus infection and transmission by mosquitoes, and 3) are there constraints to CHIKV evolution in mosquitoes? We use oral infection of *Ae*. *aegypti* mosquitoes originating from Los Angeles, California to demonstrate high laboratory transmission competence of CHIKV. We also show that oral infection of mosquitoes with CHIKV variants that produce more diverse populations are less able to infect mosquitoes than wildtype CHIKV populations. Lastly, our study provides evidence of genome-wide and regional constraints to CHIKV evolution within *Ae*. *aegypti* mosquitoes. Our results will inform public health risk assessments for potential CHIKV introduction in southern California and advance our understanding of the role of mosquitoes in CHIKV evolution.

## Introduction

Geographic expansion and explosive epidemics of chikungunya virus (CHIKV; *Togaviridae*, *Alphavirus*) over the past two decades have established it as a globally resurgent mosquito-borne pathogen. During the 2013–2017 CHIKV epidemic in the Americas, there were more than 2.5 million suspected and confirmed cases [1]. Atypical for mosquito-borne viruses, CHIKV epidemics often have high attack rates upwards of 75% [2–5]. Most symptomatic patients experience debilitating joint and muscle pain [3,4,6–8]. While rarely fatal, the persistent, incapacitating illness caused by CHIKV can have substantial economic impacts at an individual and state-level [9,10]. With no commercially available vaccine, intervention efforts during outbreaks focus on preventing mosquito exposure and mosquito population abatement.

In urban settings, the primary mosquito vectors for CHIKV are *Aedes aegypti* and, depending on the virus lineage, *Ae*. *albopictus*. Prior to 2005, *Ae*. *aegypti* was considered the primary urban vector, but a 2005–2006 Indian Ocean epidemic was associated with a rise in transmissibility by *Ae*. *albopictus*, especially in Reunion Island where *Ae*. *aegypti* were not present [11]. Adaptation to *Ae*. *albopictus* was enabled by an alanine to valine substitution in the envelope protein 1 (E1 A226V) of East, Central, South African (ECSA) lineage CHIKV [12,13], and additional ‘second-step’ point mutations in E2 that complement E1 A226V in *Ae*. *albopictus* were later characterized and likely perpetuated other outbreaks in Southeast Asia [14,15]. Due in part to these mutations, CHIKV arising from the Indian Ocean outbreak formed a new clade within the ECSA lineage, designated the Indian Ocean sublineage (IOL) [16–18]. The CHIKV epidemics in the Americas since 2013 resulted from the introduction of Asian lineage CHIKV that lacks E1 A226V [19]. This demonstrated that even in the absence of the E1 mutation, Asian lineage CHIKV can be spread efficiently in outbreaks, usually by highly abundant *Ae*. *aegypti* mosquitoes as was observed in the Americas [20–25]. In the southeastern United States, both *Aedes* spp. are endemic [26] and are competent vectors for CHIKV, as demonstrated by laboratory vector competence studies with mosquitoes from Florida [23,25] and transient local transmission of CHIKV in Florida in 2014 [27] and Texas in 2015 [28]. In California, breeding populations have persisted since 2001 for *Ae*. *albopictus* and 2013 for *Ae*. *aegypti* despite active intervention efforts [29–31]. As the result of separate introduction events, *Ae*. *aegypti* populations in northern and southern California are genetically distinct. Northern Californian populations are closely related genetically to *Ae*. *aegypti* from the south central and southeastern USA, whereas the southern California populations have greater genetic relatedness to *Ae*. *aegypti* from the southwestern USA and northern Mexico [32]. As of yet, the vector competence of either Californian *Ae*. *aegypti* population for CHIKV has not been determined and, thus, their potential risk to public health is unknown.

For CHIKV to be transmitted by a mosquito, it must overcome both anatomical and immunological barriers to establish infection, dissemination, and then transmission [33–36]. Entry and egress in the midgut and salivary glands are barriers to infection and transmission that can drastically alter the diversity and composition of intra-vector virus populations via genetic bottlenecks [37,38]. Following bottlenecking events, intra-vector population diversity is restored by population expansion in infected tissues [39,40]. Re-establishing a diverse population may be advantageous for the virus to enhance adaptability to subsequent selective pressures, where a diverse population is more likely to contain a variant that can overcome a new pressure. Supporting this hypothesis, minority variants in intra-vector virus populations have been shown to affect the fitness of West Nile virus (WNV), a mosquito-borne flavivirus [41,42]. For CHIKV, artificial removal of one host type from the alternating vertebrate-mosquito-vertebrate cycle via serial passage in mosquito cell culture resulted in expanded population diversity and greater adaptability to novel selection pressures [43]. Furthermore, Stapleford *et al*. demonstrated that the aforementioned *Ae*. *albopictus*-adaptive CHIKV mutation, E1 A226V, can quickly rise from minority to majority variant after only one or two transmission cycles in mosquitoes [44]. These studies show that the composition of the CHIKV population is important for determining infection and transmission success during mosquito infection. Whether the overall diversity of intra-vector CHIKV population affects its ability to traverse the barriers to infection, dissemination, and transmission is unknown.

Fidelity variant viruses produce populations with altered diversity and, therefore, are useful tools to study the phenotypic effects of intra-vertebrate or intra-vector virus diversity. Point mutations in the non-structural 4 (nsP4) and nsP2 proteins of CHIKV have been previously demonstrated to alter the diversity of CHIKV populations [45–48]. As part of the CHIKV replication complex, the nsP2 helicase domain unwinds the nascent RNA from the template strand and nsP4 is the RNA-dependent RNA polymerase that copies the template strand. Although the mechanism remains unclear, a nsP4 C483Y substitution (P4) was shown to produce less diverse populations in mammalian cell culture and replicate to lower titers in mosquito bodies than parental wildtype virus (WT), but the diversity of intra-mosquito populations was not characterized [46]. The combination of nsP4 C483Y and nsP2 G641D substitutions (P2P4) on the same genome had additive effects in cell culture with an additional reduction in population diversity compared to WT or each point mutant expressed alone [48]. The phenotype of P2P4 has not been assessed in mosquitoes. Recently, we reported that P4 and P2P4 (“HiFi” and “DM HiFi”, respectively), elicit more severe disease and, in contrast to previous studies that used younger mice [46,48], produce more diverse intra-host populations in an adult mouse model of CHIKV arthritis [45]. Despite the uncertainty regarding fidelity phenotypes for CHIKV P4 and P2P4, they reliably produce populations with altered diversity relative to WT and, therefore, retain utility for infection experiments evaluating the role of virus population diversity. Without prior evidence for a reduction in diversity for P4 and P2P4 in mosquitoes, we hypothesized that P4 and P2P4 would produce virus populations with enhanced diversity relative to WT due to our previous observation of enhanced diversification of both variants in mice.

In the present study, we orally infected mosquitoes with CHIKV WT, P4, or P2P4 and monitored infection at early and late timepoints. The goals of the study were three-fold: 1) to determine the laboratory vector competence of *Ae*. *aegypti* mosquitoes from Los Angeles, California; 2) to evaluate the effect of altered intra-vector diversity on mosquito infection, dissemination, and transmissibility; 3) to characterize the evolutionary dynamics of CHIKV populations in mosquitoes. Due to the previous demonstration of high laboratory vector competence of *Ae*. *aegypti* from southern Mexico [25,49] and the southeastern USA for CHIKV [23,25], we hypothesized that high competence for CHIKV transmission would also be observed in *Ae*. *aegypti* from Los Angeles, California. Based on our previous observation of enhanced diversification of P4 and P2P4 CHIKV in mice [45] and the prior evidence of attenuated replication for P4 in mosquito bodies [46], we predicted that both variants would produce more diverse populations and replicate more poorly than WT in mosquitoes, resulting in lower rates of infection in mosquito bodies. Lastly, we expected to observe purifying selection of CHIKV populations in mosquito bodies following midgut escape, as was seen with the related alphavirus, Venezuelan equine encephalitis virus [39]. We additionally report sequence conservation in CHIKV non-coding regions across mosquitoes, providing evidence suggesting their functional importance during mosquito infection.

## Materials and methods

### Viruses and cells

Generation of infectious DNA clones of IOL CHIKV outbreak strain 06–049 (GenBank accession number AM258994.1) with or without mutagenesis of nsP4 C483Y and nsP2 G641D by site-directed mutagenesis was previously described [43,45,46]. Full-length CHIKV RNA was *in vitro* transcribed (IVT) from infectious clones (mMESSAGE mMACHINE SP6 transcription kit; Thermo Fisher Scientific). Virus particles were rescued by electroporating 5 μg of IVT RNA into baby hamster kidney cells (BHK-21; ATCC CCL-10) and harvesting cell culture supernatant 48 hours post-electroporation, as previously described [43]. Virus generated previously from the infectious CHIKV clone used here exhibited comparable replication kinetics to the parental isolate [43], supporting its use in assessing vector competence. BHK-21 cells and African green monkey cells (Vero; ATCC CCL-81) used for virus titrations were maintained in high-glucose Dulbecco’s modified Eagle medium (DMEM; Gibco, Thermo Fisher Scientific) supplemented with 10% fetal bovine serum (FBS; Gibco, Thermo Fisher Scientific) and 1% penicillin-streptomycin (Gibco, Thermo Fisher Scientific) at 37°C and 5% CO_2_.

### Mosquitoes

*Ae*. *aegypti* mosquitoes were initially field-collected as larvae in 2016 by the Los Angeles County Mosquito and Vector Control District and identified morphologically. Wild females were bloodfed in the laboratory and eggs were subsequently collected from the bloodfed females. Wild adults were killed and tested individually for Zika, dengue and chikungunya viruses by quantitative RT-PCR (qRT-PCR) using previously described primer sets and cycle parameters [50–52] with the SensiFAST Probe Lo-ROX One-Step kit (Bioline, Memphis, TN) on a ViiA 7 instrument (Thermo Fisher Scientific). After verifying all adults tested negative for those 3 viruses, eggs were hatched and used to generate a colony. The genotypic identity of selected individuals from the colony was confirmed by partial sequencing of the cytochrome B gene [53] and cross-referenced to the *Ae*. *aegypti* genome. Colony size was maximized by hatching eggs from multiple females at each generation to prevent genetic bottlenecking. All mosquito infections were performed with 4 to 7-day old 12^th^-generation (F12) *Ae*. *aegypti* mosquitoes.

### Oral infection of mosquitoes

Oral exposure of mosquitoes to CHIKV was performed in two replicate experiments. In experiment 1, cohorts of mosquitoes were exposed to either WT, P4, or P2P4. In experiment 2, additional cohorts of mosquitoes were exposed to either WT or P2P4. Mosquitoes were held at 26°C and 80% relative humidity with a 12:12 hour light:dark cycle inside cardboard pint cartons. On day 0, mosquitoes were provided artificial infectious bloodmeals heated to 37°C in a membrane feeder apparatus (Hemotek Ltd, Blackburn, UK) utilizing a thinly stretched parafilm membrane (Parafilm M, Bemis). Bloodmeals consisted of 3 parts heparinized sheep blood (Hemostat Laboratories, Dixon, CA) and 1 part electroporated virus stock diluted in phosphate-buffered saline such that the virus concentration in the bloodmeal was approximately 7.3 log_10_ plaque-forming units (pfu) per mL. Bloodmeals were offered to each container of mosquitoes for 20 minutes. Each bloodmeal was back-titrated by plaque assay to verify matched infectious virus titers across CHIKV variants.

After bloodfeeding, mosquitoes were cold-anesthetized and those with absent or partial abdominal distention were discarded. Bloodfed mosquitoes were provided 10% sucrose solution *ad libitum* for the duration of the experiment. At days 5 and 12 post-feeding (dpf), subsets of mosquitoes from each treatment group were cold-anesthetized to allow collection of legs and wings (L/W), saliva, and bodies. Saliva was collected for 15 minutes in capillary tubes containing 5 μL FBS [54]. Contamination between samples during mosquito dissections was minimized by using 8 forceps and cleaning the forceps after handling each body, L/W, or capillary tube. Forceps were washed in nuclease-free water (Ambion, Thermo Fisher Scientific) and 70% ethanol solution, and then wiped dry with a clean paper towel. Post-salivation, capillary tubes were placed in 2 mL microcentifuge tubes with 0.5 mL DMEM and then centrifuged at 10,000 rpm for 1 minute to remove the saliva from the capillary tube. Bodies and L/W stored in 2 mL microcentrifuge tubes with 0.5 mL DMEM and a 4 mm diameter glass bead were homogenized by shaking for 4 minutes at 30 shakes/second in a Qiagen Tissuelyser.

### CHIKV RNA extraction and titration

For titration of infectious virus particles in bloodmeals and saliva, plaque assays were performed with duplicate serial dilutions of 125 μL inoculated on Vero cell monolayers as previously described [55]. For titration of CHIKV RNA genome copies (gc), CHIKV RNA was extracted from 200 μL of each mosquito tissue homogenate and bloodmeal. Extractions were performed with a MagMax-96 viral isolation kit (Thermo Fisher Scientific) on a MagMax Express-96 particle processer (Thermo Fisher Scientific). All extracted RNA samples were stored at -80°C. Extracted RNA was titrated in triplicate by qRT-PCR with previously described primers (CHIKV 6856, 6981, and 6919-FAM) [51] on QuantStudio 5 instruments (Thermo Fisher Scientific). Serially diluted CHIKV IVT RNA was used for standard curves. For a sample to be treated as a positive detection, all three qRT-PCR replicates were required to be positive (C_t_<40). Biologically discordant qRT-PCR positive results (e.g., CHIKV RNA detected in L/W, but not body) were infrequent (44/735 samples tested) and always low CHIKV RNA titer (S1 Fig), and thus were treated as false positives and excluded from the analyses. The limit of detection was 2 plaque-forming units (PFU) per sample for plaque assays and 25 gc per sample for qRT-PCR.

### Amplicon library preparation and Illumina sequencing

Amplicon libraries were prepared as previously described [45]. Briefly, CHIKV RNA isolated from bodies and saliva was amplified by nine separate RT-PCR reactions using a high-fidelity RT-PCR kit and the primers described in Riemersma et al., 2019 [45]. The nine overlapping cDNA amplicons spanning from the 5’ to 3’ untranslated regions (UTR), excluding the first 14 nucleotides of the 5’ UTR and the last 55 nucleotides of the 3’ UTR, were pooled at equimolar ratios and enzymatically fragmented to approximately 150 bp. Illumina sequencing libraries were prepared with the fragmented cDNA using a NEBNext Ultra II DNA library prep kit and NEBNext Multiplex Oligos (New England Biolabs). Libraries of P4 infectious plasmid DNA with (pY_PCR) and without (pY) RT-PCR amplification, and P4 *in vitro* transcribed RNA were used as sequencing controls. An unrelated library (WNV cDNA) was also included as a control for index hopping with Illumina HiSeq 4000 sequencing [56]. All libraries were sequenced in parallel with paired-end 150 reads on a single flow cell lane of an Illumina HiSeq 4000 instrument at the UC Davis DNA Technologies Core. Raw fastq files are available from NCBI Sequence Read Archive under BioProject entry PRJNA541092.

### Paired-end read processing and bioinformatics

Demultiplexed reads were quality (>Q35) and adapter-trimmed with Trimmomatic (v0.36) [57] and then RT-PCR primer-trimmed with cutadapt (v1.16) [58]. Paired-end reads with at least 50 bp of overlap were then merged and non-overlapping regions were trimmed with BBMerge [59]. Paired reads with any discrepancy in base calls in the overlapped region, or with overlapping regions less than 50 bp were discarded. Reference-guided alignment of merged reads was performed with the Burrows Wheeler alignment tool (bwa mem, v0.7.5). To control for variance in depth of sequencing between samples, reads were randomly down-sampled with BBTools (DOE Joint Genome Institute). Single nucleotide variants (SNV) were called by LoFreq* (v2.1.2) [60] and annotated by SNPdat (v1.0.5) [61]. Base counts from aligned reads at each genome position were generated with pysamstats (https://github.com/alimanfoo/pysamstats) and used as an input for in-house R scripts to calculate Shannon entropy (SE) and specific nucleotide substitution frequencies. Nucleotide diversity (π) at all sites was calculated by SNPgenie (v1.2) [62]. A minimum coverage threshold of 200 was used for all analyses. The in-house Bash and R scripts used are available at https://github.com/kasenriemersma/CHIKV-NGS-diversity.

### Sequencing data analyses

CHIKV population diversity was characterized by four metrics: mean nucleotide diversity (π) and Shannon entropy (SE) across all sites, substitution frequency per 10,000 nucleotides sequenced, and abundance of single-nucleotide variants (SNV). Nucleotide diversity is the mean number of pairwise nucleotide differences per site among all sequences and is not biased by the distribution of allele frequencies [63,64]. On the other hand, SE estimates the genetic ‘uncertainty’ within a virus population and is biased by the distribution of allele frequencies [64,65]. For virus populations, where low-frequency polymorphisms typically far outnumber high-frequency polymorphisms, SE captures the degree of low-frequency variance. Where nucleotide diversity and SE describe population variance independent of a reference sequence, substitution frequency was calculated as the number of nucleotide differences relative to the reference infectious clone sequence. Substitution frequency was used to estimate divergence of intra-mosquito CHIKV populations from the consensus CHIKV sequence in the bloodmeals.

To evaluate the distribution of mutations across the genome, a sliding window approach was applied to quantify the regional abundance of polymorphisms. The mean number of polymorphisms per site was quantified in 60-nucelotide windows in 20-nucleotide steps spanning from the 5’ UTR to the 3’ UTR. A maximum of 3 polymorphisms are possible per site (e.g., for reference nucleotide A: A>U, A>C, and A>G). Mean polymorphism abundance was evaluated on per-window and per-genome element bases. For the per-window analysis, upper and lower cut-offs were defined by the genome-wide mean abundance +/- 2 standard deviations. Regions above the upper cut-off were designated as mutational hot-spots and regions below the lower cut-off were designated as cold-spots. For the per-genome element analysis, windows were binned by gene or non-coding region and the mean values per bin were compared to the genome-wide mean.

### Statistical analyses

All statistical analyses were performed with GraphPad Prism 8 software (GraphPad Software, CA, USA). Statistical significance was assigned to *p*-values less than 0.05.

## Results

### *Ae*. *aegypti* from Los Angeles, CA, USA are competent vectors of Indian Ocean lineage CHIKV

**Fig 1:**
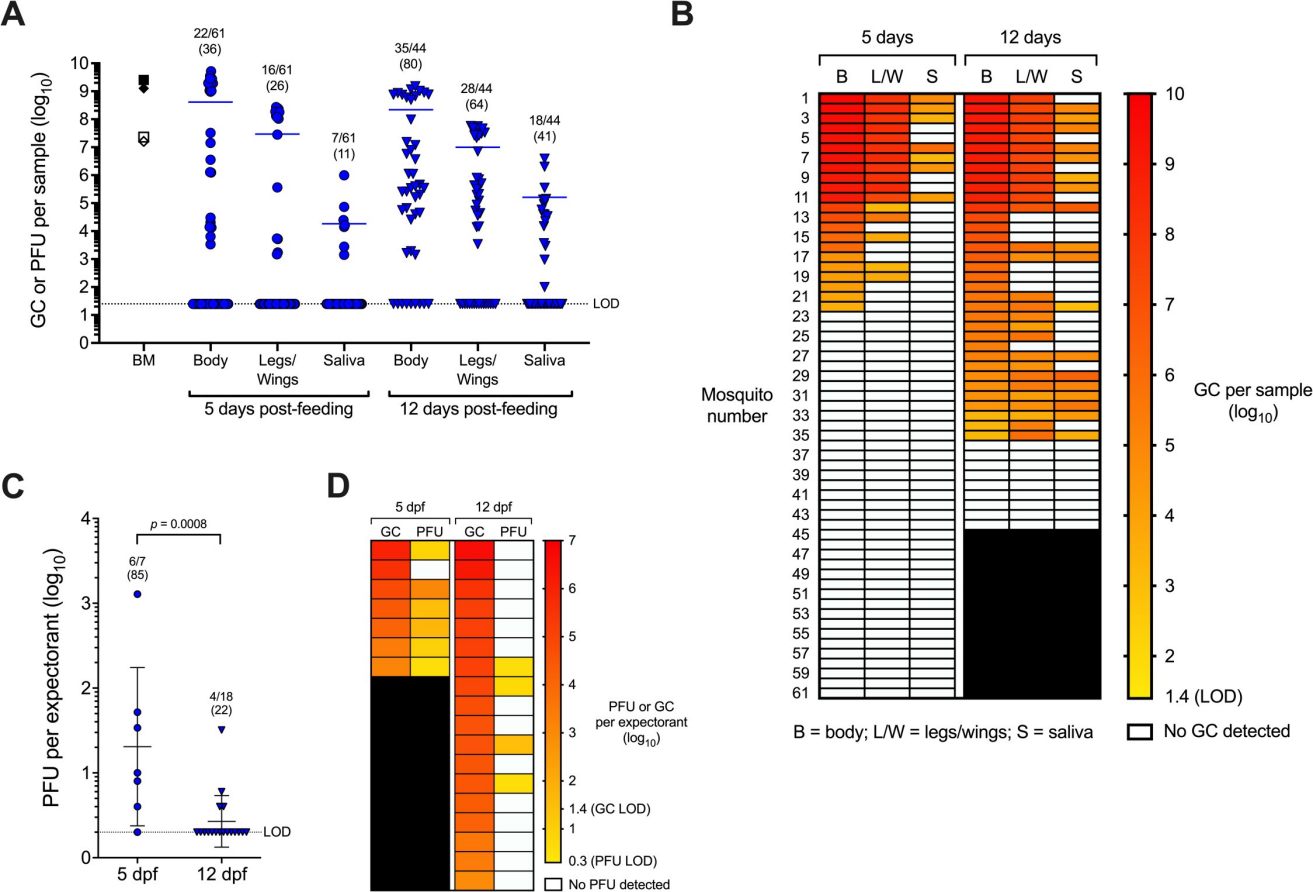
Vector competence of *Aedes aegypti* from Los Angeles for Indian Ocean Lineage CHIKV. A) Viral RNA and infectious virus titers in bloodmeals (BM) and mosquito samples at 5 or 12 days post-feeding. Open symbols = CHIKV plaque-forming units (PFU), closed symbols = CHIKV genome copies (GC). For BM, symbol shapes represent replicate experiments. For mosquito samples, each symbol represents an individual mosquito, lines represent mean titers, and proportions of positive samples from total bloodfed mosquitoes are shown with percentages (in parentheses) above groups. B) Heatmap of GC per sample arranged by individual mosquitoes. C) PFU detected per expectorant at 5 or 12 dpf. D) Heatmap of GC and PFU per expectorant arranged by individual mosquitoes. Lines represent mean titers. LOD = limit of detection.

*Ae*. *aegypti* mosquitoes (F12) from Los Angeles were orally exposed to IOL WT CHIKV via artificial bloodmeal in two replicate experiments. Artificial bloodmeal titers for the replicate experiments were well-matched at 9.4 gc/mL and 7.3 pfu/mL for experiment 1, and 9.1 log_10_ gc/mL and 7.2 log_10_ pfu/mL for experiment 2 (Fig 1A). To determine vector competence, rates of infection, dissemination, and transmission were assessed at 5 and 12 dpf. Infection rates were determined by the number of CHIKV RNA-positive bodies in all orally exposed mosquitoes per group. Dissemination rates were determined by the number of CHIKV RNA-positive L/W samples from bloodfed mosquitoes. Transmission rates were determined by the number of CHIKV RNA-positive saliva samples from bloodfed mosquitoes. At 5 dpf, infection, dissemination, and transmission rates determined by CHIKV RNA detection were 22/61 (36%), 16/61 (26%), and 7/61 (11%) respectively (Fig 1A and 1B). At 12 dpf, infection, dissemination, and transmission rates determined by CHIKV RNA detection were 35/44 (80%), 28/44 (64%), and 18/44 (41%), respectively (Fig 1A and 1B). Mean CHIKV RNA titers (log_10_-transformed) in bodies, L/W, and saliva were similar between days 5 and 12 (two-way ANOVA, *p*-values>0.05). In contrast, the infection, dissemination, and transmission rates were significantly greater at day 12 compared to day 5 (Fisher’s exact tests, *p*-values<0.0009). To assess transmission of infectious CHIKV, plaque assays were performed on saliva expectorants positive for CHIKV RNA. Higher levels of infectious CHIKV were transmitted at 5 dpf (mean 1.3 log_10_ PFU) than 12 dpf (mean 0.4 log_10_ PFU) (Fig 1C, Kruskal-Wallis test with Dunn’s post-hoc, *p* = 0.0008). Although the number of mosquitoes in the 5 dpf cohort was low, infectious virus was also detected in a higher proportion of expectorants at 5 dpf (6/7, 85%) than 12 dpf (4/18, 22%). There was no association between RNA and infectious virus titers (Fig 1D), which may be explained by the stochastic nature of low titer samples that may not have even distributions of infectious CHIKV particles in solution. These results demonstrate that *Ae*. *aegypti* mosquitoes originating from Los Angeles are competent vectors for CHIKV.

### Fidelity variant CHIKV exhibit attenuated infectivity in Ae. aegypti from Los Angeles, CA, USA

**Fig 2:**
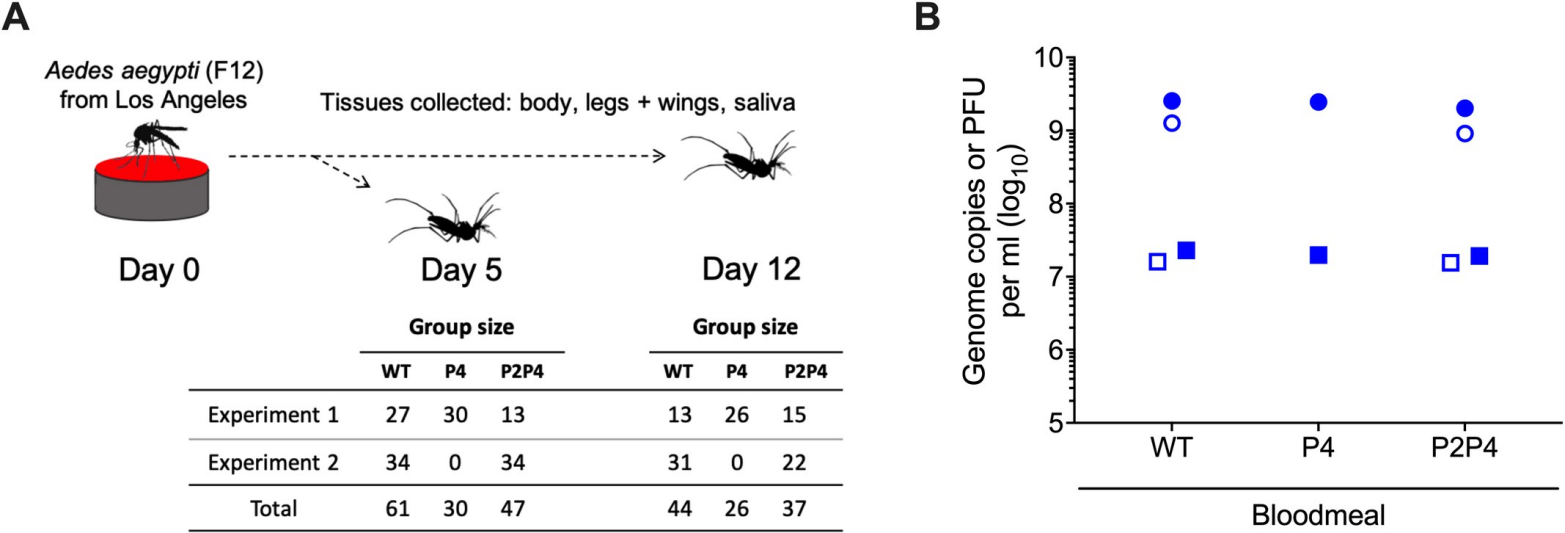
Mosquito infection experiment design and artificial bloodmeal titers for exposure of Los Angeles *Ae*. *aegypti* to CHIKV WT and fidelity variant P4 and P2P4. A) Experiment design with group sizes for each treatment group. F12 = 12^th^ generation. B) Back-titration of bloodmeals demonstrating matched CHIKV RNA and infectious virus titers. Closed symbols = replicate experiment 1; open symbols = replicate experiment 2. Circles = genome copies per ml; squares = plaque-forming units (PFU) per ml. Bloodmeal titers for WT presented in panel B are the same as presented in Fig 1A.

To evaluate the effects of intra-vector CHIKV diversity on infection dynamics, *Ae*. *aegypti* mosquitoes were orally exposed to WT, P4, or P2P4 via artificial bloodmeal in two replicate experiments (Fig 2A). Artificial bloodmeal titers of CHIKV RNA and infectious virions for WT, P4, and P2P4 were well-matched across groups and replicate experiments (Fig 2B). RNA titers for WT, P4, and P2P4 in the first replicate experiment were 9.4, 9.4, and 9.3 log_10_ gc/mL and 7.4, 7.3, and 7.3 log_10_ pfu/mL, respectively. For the second replicate experiment, WT and P2P4 bloodmeal titers were 9.1 and 9.0 log_10_ gc/mL, and 7.2 and 7.2 log_10_ pfu/mL, respectively. In Fig 2B, the bloodmeal titers for WT are the same data as presented in Fig 1A.

**Fig 3:**
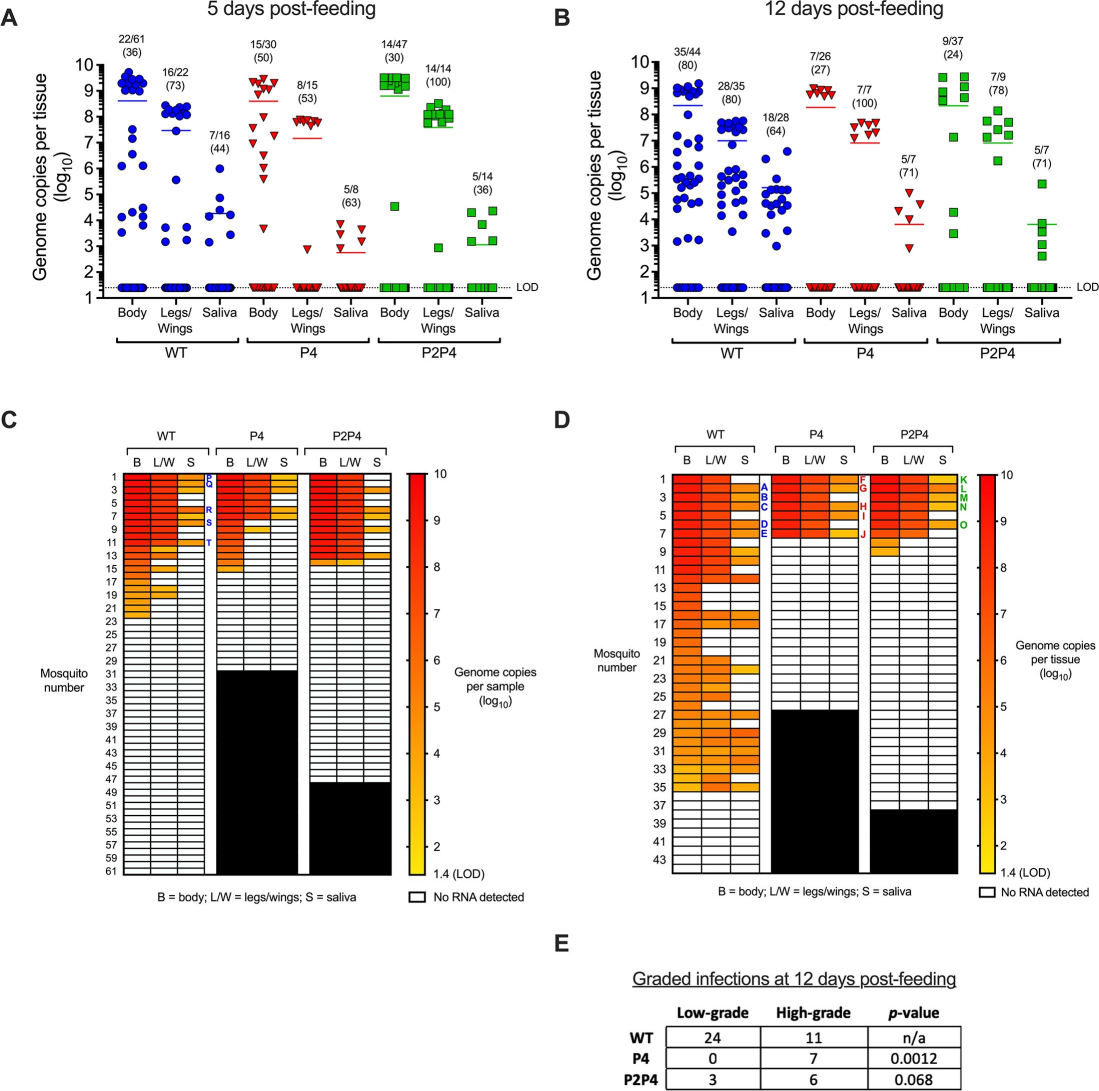
**Infection, dissemination, and transmission of WT and fidelity variant CHIKV at 5 (A and C) or 12 days post-feeding (B and D).** Data for WT are same as Fig 1. For A and B, each symbol represents an individual mosquito, lines represent mean CHIKV RNA titers, and proportions of positive samples with percentages (in parentheses) are shown above groups. For reported proportions, the denominator for legs/wings and saliva samples are the number of infected bodies and number of disseminated infections, respectively. For C and D, samples from individual mosquitoes are arranged horizontally. LOD = limit of detection. Individual mosquito samples selected for sequencing are labeled in C and D with corresponding mosquito identifying letters A to T. E) Infected bodies at 12 days post-feeding were designated as low-grade (<8 log_10_ genome copies) or high-grade (>8 log_10_ genome copies). Proportions of low-grade infections for P4 and P2P4 were compared against WT by Fisher exact test, *p*-values are listed. The data for WT-infected mosquitoes presented here are the same as presented in Fig 1A and 1B.

All three CHIKV variants infected, disseminated, and transmitted in *Ae*. *aegypti* by 5 dpf (Fig 3A and 3C). Infection rates were determined from the number of CHIKV RNA-positive bodies in all orally exposed mosquitoes per group. To evaluate dissemination efficiency, effective dissemination rates were determined by the number of CHIKV RNA-positive L/W samples from infected mosquitoes. Similarly, to evaluate transmission efficiency, effective transmission rates were determined by the number of CHIKV RNA-positive saliva samples from mosquitoes with disseminated infections. There were no significant differences observed in infection, effective dissemination, or effective transmission rates between variants at 5 dpf (Table 1). Transmission was also observed in mosquitoes exposed to each variant on 12 dpf (Fig 3B and 3D). Infection rates at 12 dpf were significantly higher for WT (80%) than P4 (27%) or P2P4 (24%) (Fig 3B and Table 1; Fisher’s exact tests, *p*-values<0.0001). No statistically significant differences in effective dissemination or transmission were observed at 12 dpf (Table 1). For Fig 3, the data presented for WT-infected mosquitoes are the same as in Fig 1A and 1B.

**Table 1:** Infection rates, and effective dissemination (CHIKV RNA-positive L/W samples from infected mosquitoes) and transmission (CHIKV RNA-positive saliva samples from mosquitoes with disseminated infections) rates of CHIKV WT and P4 and P2P4 fidelity variants in *Ae*. *aegypti* from Los Angeles, California at 5 and 12 days post-feeding (DPF). Within each day, rates for P4 and P2P4 were compared versus WT by Fisher exact test and *p*-values are shown. Statistically significant values are highlighted in bold font.

Virus	DPF	Infection		*p*-value	Dissemination		*p*-value	Transmission		*p*-value
WT	5	36%	(22/61)	n/a	73%	(16/22)	n/a	44%	(7/16)	n/a
P4	5	50%	(15/30)	0.56	53%	(8/15)	0.3	63%	(5/8)	0.47
P2P4	5	30%	(14/47)	0.54	100%	(14/14)	0.063	36%	(5/14)	0.72
WT	12	80%	(35/44)	n/a	80%	(28/35)	n/a	64%	(18/28)	n/a
P4	12	**27%**	**(7/26)**	**<0.0001**	100%	(7/7)	0.33	71%	(5/7)	0.99
P2P4	12	**24%**	**(9/37)**	**<0.0001**	78%	(7/9)	0.99	71%	(5/7)	0.99

We next assessed whether the magnitude of CHIKV infection varied in mosquitoes exposed to the different variants. To assess whether the distribution of high- and low-grade infections varied between variants, infected bodies were assigned as either high-grade (>8 log_10_ gc) or low-grade (<8 log_10_ gc). Log-transformed CHIKV RNA titers for infected bodies at 5 and 12 dpf fit a bimodal distribution with tightly clustered high-titer samples and broadly distributed low-titer samples (Fig 3A and 3B). WT-infected mosquitoes developed a higher proportion of low-grade infections in bodies at 12 dpf than P4 or P2P4-infected mosquitoes (Fig 3E; Fisher’s exact tests, *p* = 0.0012 and 0.068, respectively). Of the low-grade infections at 5 and 12 dpf, 33% (11/33) of WT-infected mosquitoes were transmission-competent, whereas 0% of P4 (0/8) and P2P4-infected mosquitoes (0/4) were transmission-competent. These results indicate that attenuation in infectivity of P4 and P2P4 is due to reduced ability to overcome the midgut infection barrier. This attenuation ultimately reduces transmissibility, since high-grade infections are required to transmit P4 and P2P4 CHIKV.

### Fidelity variant CHIKV diversifies more than wildtype CHIKV in mosquitoes

**Fig 4:**
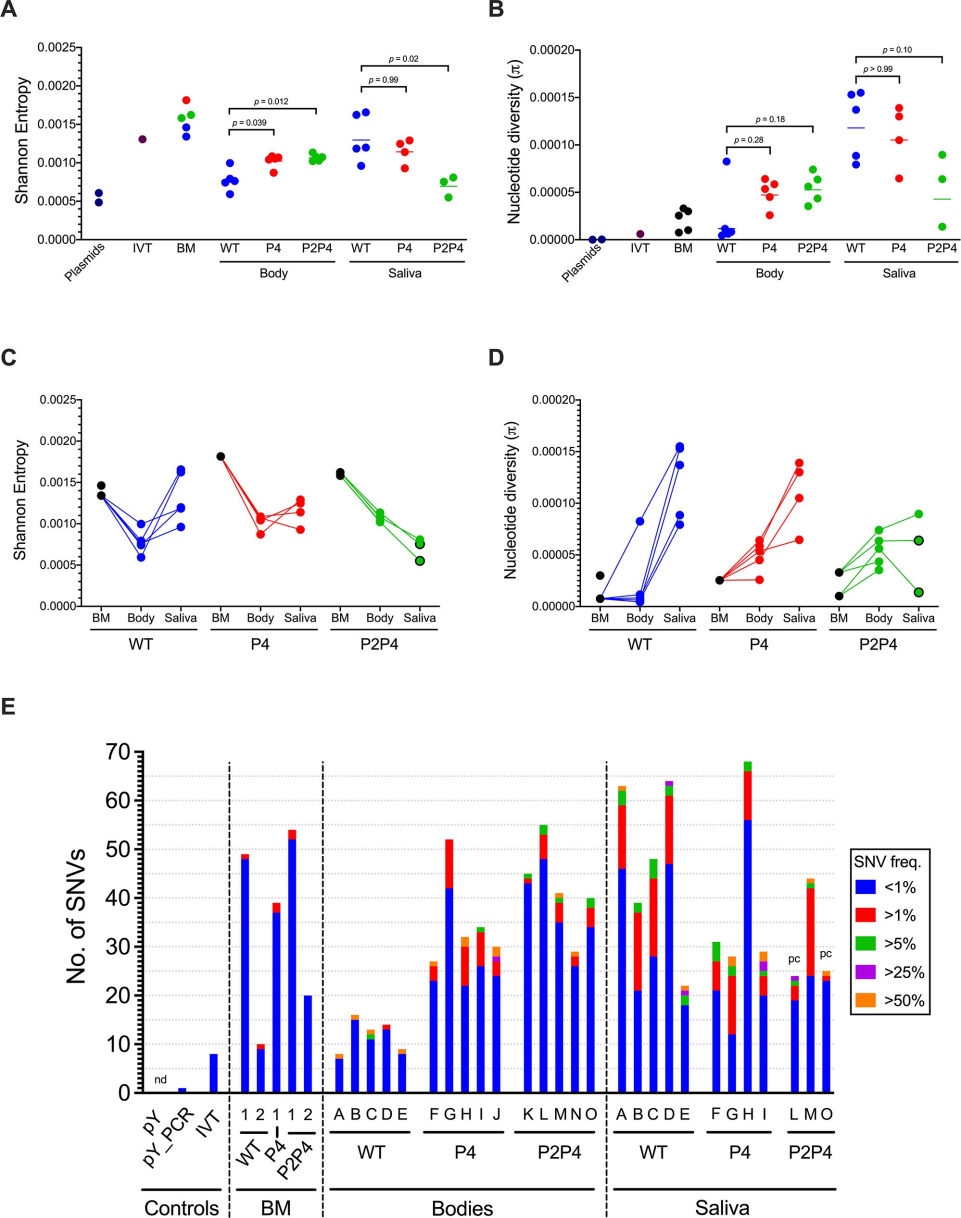
Fidelity variant CHIKV P4 and P2P4 diversify more than wildtype in mosquito bodies. A) Mean per-site Shannon entropy of CHIKV populations. B) Nucleotide diversity of CHIKV populations. For A and B, treatment group mean values are represented by lines and were compared to WT by Kruskal-Wallis test, *p*-values reported. pY = plasmid DNA, pY_PCR = plasmid DNA with PCR, IVT = *in vitro* transcribed RNA, BM = bloodmeal. Data presented in A and B are reproduced in C and D to show within-mosquito trends. E) Single nucleotide variant (SNV) abundance in bodies and saliva at 12 days post-feeding, with bloodmeals and sequencing controls included. SNV freq. = SNV frequency; pc = partial genome sequencing coverage; nd = none detected.

We detected evidence of ample low-level variance in all five bloodmeals, demonstrated by comparably higher SE values than nucleotide diversity (Fig 4A and 4B) and abundant SNVs at <1% frequency (Fig 4E). Similar trends were observed for CHIKV IVT RNA but not the pY or pY_PCR controls, indicating that the variance observed in the bloodmeals is likely due to mutations generated during *in vitro* transcription of infectious clones prior to virus rescue in BHK-21 cells. The number of SNVs (49, 39, and 52) detected in the three bloodmeals from experiment 1 was higher than the bloodmeals from experiment 2 (10 and 20) (Fig 4E). In-depth analysis of frequencies of specific mutations revealed that this difference between bloodmeals was due primarily to elevated G>U substitution frequencies in experiment 1 bloodmeals (S3 and S6 Figs). Importantly, the G>U bias was not observed in mosquito bodies or saliva (S4–S6 Figs), indicating the issue was limited to certain bloodmeals and not due to sequencer-derived error. Transversion substitutions like G>U can be induced by oxidation of guanine bases by reactive oxygen species (ROS) found in blood [66–68], or by acoustic shearing during next generation sequencing (NGS) library preparation [69,70]. Since the bloodmeals for both experiments were created from the same virus stocks and acoustic shearing was not performed, we suspect the observed G>U bias was caused by oxidative damage from ROS in experiment 1 bloodmeals.

Five mosquitoes with high CHIKV RNA titers in bodies on 12 dpf were selected from each treatment group for deep sequencing of bodies and saliva to characterize virus populations. Saliva titers for one P4-infected mosquito and two P2P4-infected mosquitoes were inadequate (2.9, 2.6 and 3 log_10_ gc per expectorant) for RT-PCR amplification prior to library preparation and were therefore excluded. In addition to the CHIKV libraries prepared from mosquitoes, libraries prepared for the bloodmeals from both experiments, CHIKV infectious plasmid DNA (pY), pY with PCR amplification (pY_PCR) to control for mutational error introduced by PCR, CHIKV IVT RNA to control for mutational error introduced by IVT during preparation of the infectious clone derived virus stocks, and WNV cDNA to control for index hopping during sequencing were also sequenced in parallel. In the WNV cDNA library, 0.6% of processed reads aligned to the CHIKV reference genome, indicating that the magnitude of index hopping between libraries was small and is unlikely to have biased our analyses. The mean depth of coverage post-processing across all libraries ranged from 3,570 to 5,229 reads (Table 2). For two P2P4 saliva samples (highlighted in Fig 4E by ‘pc’), only partial genome coverage was achieved with 86.8% and 86.9% of the genome covered by at least 200 reads (Table 2). All other samples had at least 99.0% of the genome covered by at least 200 reads (Table 2). Due to incomplete coverage of the UTRs by the RT-PCR primers, the maximum genome coverage possible for mosquito samples was 99.4% The fidelity-altering point mutations in *nsP2* and *nsP4* were stable with no evidence of reversion in any sequenced sample.

**Table 2:** Descriptive statistics for Illumina NGS from experimentally CHIKV-infected *Ae*. *aegypti* mosquito bodies and saliva from Los Angeles, California. Exp = replicate experiment number; % covered = percent of reference genome covered by >200 reads; mean depth = mean depth of sequencing coverage across the genome; SE = Shannon entropy; pi = nucleotide diversity; SNV count = number of SNVs identified by variant caller; n/a = not applicable; pY = plasmid DNA; pY_PCR = plasmid DNA with PCR; IVT = *in vitro* transcribed RNA.

Day	Virus	Exp	Mosquito	Sample	% Covered	Mean depth	SE	pi	SNV count
12	WT	2	A	Body	99.3	5,221	7.92E-04	4.49E-06	8
12	WT	2	B	Body	99.3	5,209	7.42E-04	8.36E-06	16
12	WT	2	C	Body	99.3	4,882	9.96E-04	8.25E-05	13
12	WT	2	D	Body	99.2	5,221	5.92E-04	1.16E-05	14
12	WT	2	E	Body	99.3	5,147	7.63E-04	6.27E-06	9
12	WT	2	A	Saliva	99.1	4,430	1.66E-03	1.37E-04	63
12	WT	2	B	Saliva	99.1	4,688	1.20E-03	8.85E-05	39
12	WT	2	C	Saliva	99.1	4,466	1.19E-03	1.55E-04	48
12	WT	2	D	Saliva	99.1	4,005	1.62E-03	1.53E-04	64
12	WT	2	E	Saliva	99.1	4,365	9.61E-04	7.93E-05	22
12	P4	1	F	Body	99.3	5,068	1.05E-03	2.59E-05	27
12	P4	1	G	Body	99.3	4,896	1.09E-03	5.35E-05	52
12	P4	1	H	Body	99.2	5,105	1.04E-03	5.85E-05	32
12	P4	1	I	Body	99.3	5,229	8.71E-04	4.51E-05	34
12	P4	1	J	Body	99.2	4,522	1.07E-03	6.41E-05	30
12	P4	1	G	Saliva	99.2	4,571	1.14E-03	6.46E-05	31
12	P4	1	H	Saliva	99.1	4,506	1.25E-03	1.39E-04	30
12	P4	1	I	Saliva	99.1	4,805	1.29E-03	1.05E-04	68
12	P4	1	J	Saliva	99.0	3,570	9.28E-04	1.30E-04	30
12	P2P4	1	K	Body	99.1	5,077	1.07E-03	4.35E-05	45
12	P2P4	1	L	Body	99.3	4,695	1.13E-03	6.35E-05	55
12	P2P4	1	M	Body	99.2	5,030	1.08E-03	7.40E-05	41
12	P2P4	2	N	Body	99.2	4,663	1.03E-03	3.54E-05	29
12	P2P4	2	O	Body	99.1	4,992	1.02E-03	5.61E-05	40
12	P2P4	1	L	Saliva	86.8	3,589	7.53E-04	6.39E-05	24
12	P2P4	1	M	Saliva	99.1	4,900	8.09E-04	8.96E-05	44
12	P2P4	2	O	Saliva	86.9	4,814	5.50E-04	1.37E-05	25
5	WT	1	P	Body	99.2	4,016	1.09E-03	1.01E-05	13
5	WT	1	Q	Body	99.2	5,010	9.93E-04	1.20E-05	18
5	WT	1	R	Body	99.3	4,956	1.08E-03	1.21E-05	15
5	WT	2	S	Body	99.1	4,197	9.89E-04	5.59E-05	14
5	WT	2	T	Body	99.1	3,524	1.00E-03	5.30E-05	11
0	WT	1	n/a	BM	99.2	5,186	1.46E-03	3.00E-05	49
0	WT	2	n/a	BM	99.3	5,101	1.34E-03	7.56E-06	10
0	P4	1	n/a	BM	99.2	4,730	1.81E-03	2.54E-05	39
0	P2P4	1	n/a	BM	99.2	5,221	1.62E-03	3.30E-05	54
0	P2P4	2	n/a	BM	99.2	5,154	1.58E-03	1.01E-05	20
n/a	P4	n/a	n/a	pY	99.9	5,094	4.85E-04	0	0
n/a	P4	n/a	n/a	pY_PCR	99.3	5,117	6.07E-04	2.85E-07	1
n/a	P4	n/a	n/a	IVT	99.3	5,130	1.31E-03	6.08E-06	8

The sequencing controls revealed greater diversity, particularly by SE, in the IVT RNA library relative to the plasmid libraries with and without PCR (Fig 4A, 4B and 4E). The enhanced diversity in IVT RNA was reflected in the comparable diversity observed in the bloodmeals (Fig 4A, 4B and 4E) which were derived from IVT RNA. For mosquito samples, an overall trend of increased diversity compared to WT for CHIKV fidelity variant P4 and P2P4 populations was observed in mosquito bodies at 12 dpf. The mean SE values for P4 and P2P4 populations from infected bodies were elevated relative to WT populations (Fig 4A; Kruskal-Wallis test with Dunn’s post-hoc, *p* = 0.039 and 0.012, respectively). Similarly, mean nucleotide diversities were also elevated for P4 and P2P4 populations in bodies, but the differences were not statistically significant due to a single high-diversity WT population from one mosquito body (Fig 4B, Kruskal-Wallis test with Dunn’s post-hoc, *p* = 0.28 and 0.18, respectively). Although P4 and P2P4 populations were more diverse in bodies, the opposite trend was observed in saliva. WT populations from saliva exhibited comparable SE and nucleotide diversity as P4 (Fig 4A–4D; Kruskal-Wallis test with Dunn’s post-hoc, *p*>0.99) and greater SE and nucleotide diversity than P2P4 (Fig 4A–4D; Kruskal-Wallis test with Dunn’s post-hoc, *p* = 0.02 and 0.10). In terms of SNV abundance, significantly more SNVs were detected in bodies of P4 and P2P4-infected mosquitoes than in WT-infected mosquitoes (Fig 4E; Kruskal-Wallis test with Dunn’s post-hoc, *p* = 0.047 and 0.0047). Again, that trend was not shared by the populations in saliva, where there was no difference in SNV abundance between viruses (Kruskal-Wallis test, *p* = 0.48). There were no associations between RNA titer in the body or saliva and the diversity metrics (S2 Fig) indicating that the observed differences in diversity were not biased by population size.

### CHIKV diversity declines over time and maintains conserved regions in mosquitoes

**Fig 5:**
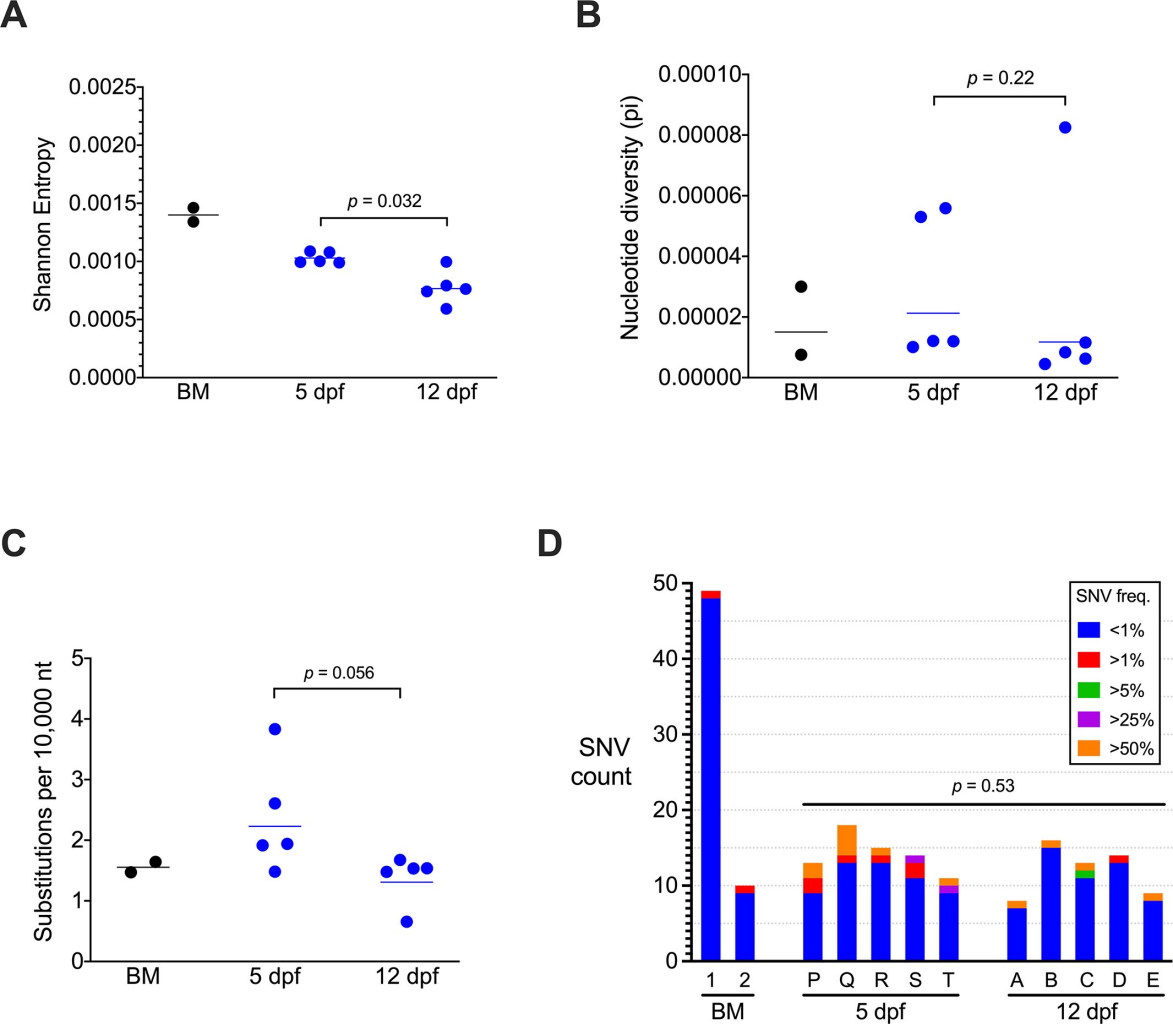
CHIKV exhibits purification of low-level population diversity over time in mosquito bodies. A) Shannon entropy, B) nucleotide diversity, and C) substitutions per 10,000 nucleotides (nt) sequenced for WT CHIKV populations in mosquito bodies at 5 or 12 days post-feeding (dpf) and bloodmeals (BM) from replicate experiments. Mean values at each day are represented by lines and were compared by Mann-Whitney tests, *p*-values are reported. D) Single nucleotide variant (SNV) abundance in bloodmeals and mosquito bodies. Letters are the corresponding mosquito identifier (Fig 1D and S1 Table). SNV freq. = SNV frequency.

To evaluate the dynamics of CHIKV populations in mosquito bodies, CHIKV populations isolated from WT-infected mosquito bodies at 5 and 12 dpf were sequenced and characterized. Full-length coverage of the coding regions was achieved for the 5 isolates from each timepoint, and the mean depth of coverage ranged from 3,524 to 5,221 reads (Table 2). Although our interpretations are based on cohorts of 5 mosquitoes sampled once at 5 or 12 dpf and not individual mosquitoes repeatedly sampled over time, purifying selection was observed for CHIKV populations in infected bodies from 5 to 12 dpf. The degree of low-level variance in CHIKV populations declined significantly (Fig 5A; Mann-Whitney test, *p* = 0.032). Furthermore, downward trends were observed for mean nucleotide diversity (Fig 5B; Mann-Whitney test, *p* = 0.22) and substitution frequency (Fig 5C; Mann-Whitney test, *p* = 0.056) although the differences were not statistically significant. Likewise, there were slightly fewer SNVs on average at 12 than 5 dpf, but the difference was not significant (Fig 5D; Mann-Whitney test, *p* = 0.53). After comparing the diversity of CHIKV populations, we next assessed trends in individual SNVs comprising each population.

Called SNVs were compared across all WT-infected bodies to identify mutations shared by at least two mosquito or bloodmeal samples. Eleven SNVs were identified as being shared by multiple mosquitoes, and all of them were low-frequency (S1 Table; μ = 0.49%, σ = 0.37%). Ten of 11 shared SNVs were non-synonymous substitutions. Although, 8 out of 11 were detected in one or both of the bloodmeals, suggesting they did not arise *de novo* in mosquitoes. One of the SNVs, E2 9357 G>U that was not detected in either bloodmeal was found at a relatively higher frequency (μ = 0.76%, σ = 0.33%) in 9 of 10 WT-infected mosquito bodies. Consensus SNVs (>50% frequency) were detected at 12 sites (none shared) across 8 mosquitoes (S1 Table). None of the consensus SNVs were detected in either bloodmeal indicating they arose *de novo* in individual mosquitoes. Unlike the shared SNVs, only 4 of the 12 consensus SNVs were non-synonymous mutations.

**Fig 6:**
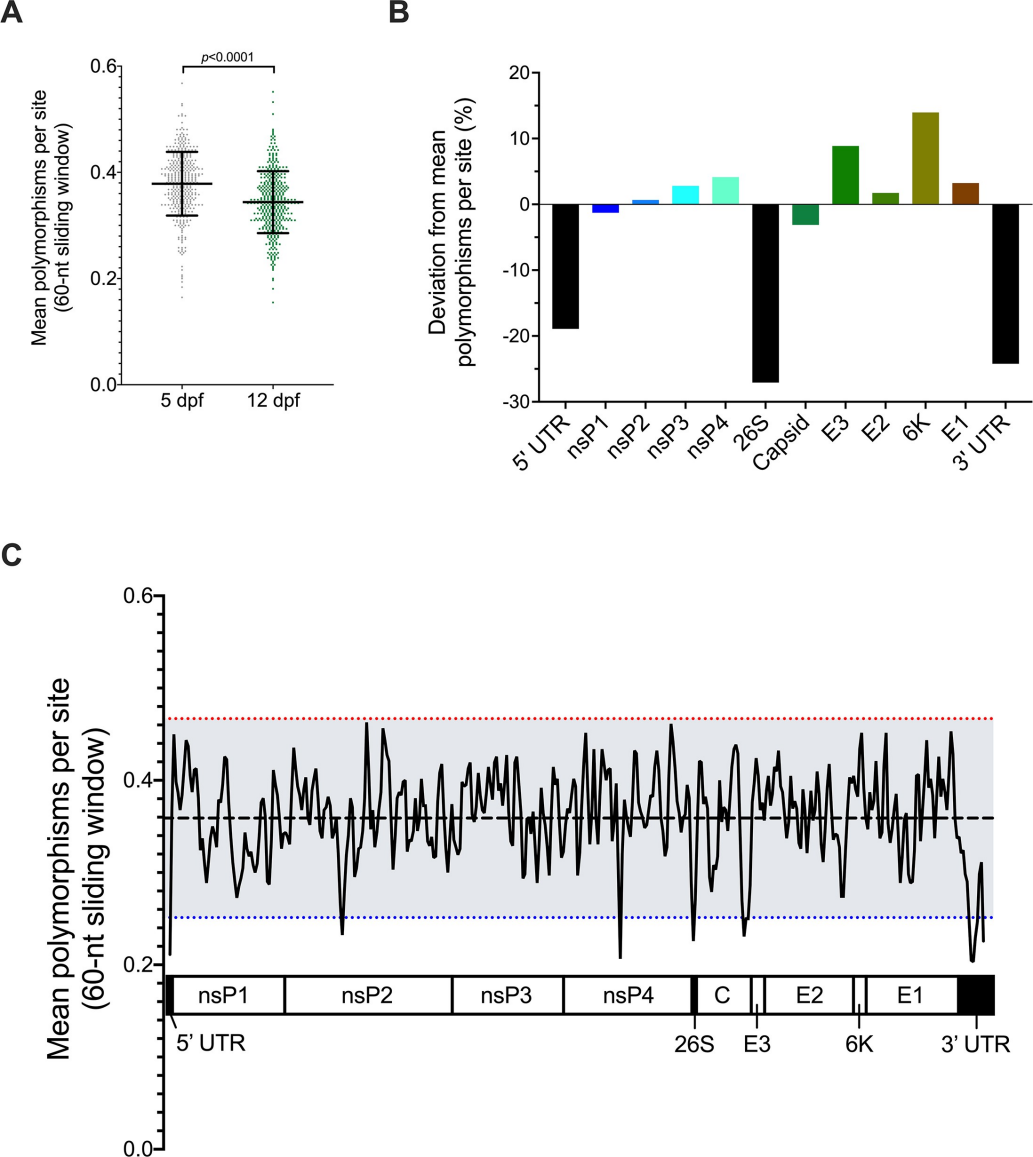
Sequence conservation in untranslated regions (UTR) identified by genome-wide sliding window analysis of mean polymorphisms per site in individual CHIKV infected mosquitoes. Number of polymorphisms per site was averaged across 60-nucleotide (nt) windows in 20-nt steps along the genome. A) Mean polymorphisms per site in all populations from 5 or 12 dpf. Each symbol represents the average of 5 populations in mosquito bodies. Total mean and standard deviation are represented by lines and error bars and were compared by paired t-test, *p*-value is reported. B) Sliding window values were averaged within each genome element and the percent deviation from the genome-wide mean is presented. C) Mean polymorphisms per site across both 5 and 12 dpf. X-axis is aligned to the CHIKV genome. Red and blue dotted lines are 2 standard deviations above the mean (dashed black line) and represent the cut-off values for mutational hot-spots and cold-spots, respectively.

After identifying individual mutations, genome-wide trends of CHIKV mutability were assessed across all WT-infected mosquitoes in combination to evaluate polymorphism abundance. The mean number of polymorphisms per site was measured in 60-nucleotide windows across the genome and regions with values 2 standard deviations above or below the mean were identified as hot- or cold-spots, respectively. In agreement with the elevated SE at 5 dpf, the mean number of polymorphisms per window was significantly higher on 5 than 12 dpf (Fig 6A; paired t-test, *p*<0.0001). Trends in polymorphism abundance were next compared between genes and non-coding regions (Fig 6B). Similar to a previous report of CHIKV evolution in *Ae*. *aegypti* cell culture [71], the structural proteins (except capsid) exhibited higher than average polymorphism abundance. Furthermore, the mean abundance in structural genes was higher than in the non-structural genes. The most conserved regions were the non-coding regions, the 5’ and 3’ untranslated regions and the 26S subgenomic promoter. This evidence of robust sequence conservation in the non-coding regions suggests they are important for CHIKV fitness in mosquitoes. Higher resolution analyses were performed next to identify hot-spot and cold-spot regions associated with greater or reduced polymorphism abundance. No hot-spot regions were detected across the genome (Fig 6C). Instead, cold-spot regions were identified in nsP2, nsP4, the 3’ end of capsid, and all three non-coding regions. The nsP2 and nsP4 cold-spot regions do not encompass the nsP2 641 and nsP4 483 fidelity-altering positions.

## Discussion

The use of *Ae*. *aegypti* mosquitoes originating from wild populations in Los Angeles, California enabled the first assessment of vector competence for CHIKV in mosquitoes from southern California. This study is also the first to characterize the evolution of CHIKV populations in mosquitoes to further our understanding of the evolutionary constraints for CHIKV in individual vectors. The results of this study support our three hypotheses: 1) *Ae*. *aegypti* from California are competent laboratory CHIKV vectors, 2) genetically diverse intra-vector CHIKV populations are attenuated, and 3) CHIKV undergoes purifying selection as it infects and disseminates in *Ae*. *aegypti*.

Laboratory vector competence of *Ae*. *aegypti* from Los Angeles, CA was demonstrated by high rates of infection (80%) and transmission (41%) 12 days after oral exposure to wildtype IOL CHIKV. Contrary to our expectations that transmission rates of infectious CHIKV increase over time to an upper limit, the transmission rate of infectious CHIKV decreased from 5 to 12 dpf (although the transmission rate of CHIKV RNA over that time increased). The temporal reduction in infectious CHIKV transmission may be explained by antiviral responses in salivary glands. With mosquito-borne flaviviruses, temporal reduction of infectious virus titers has been demonstrated in midguts [72] and salivary glands [73], with loss of infectious WNV in salivary glands associated with increased apoptosis of glandular cells, which may decrease rates of transmission as well. CHIKV-induced apoptosis has likewise been demonstrated in salivary glands [74], but a negative association between the magnitude of apoptosis and infectious titer was not reported. Additional studies are warranted to confirm the temporal decline in infectious CHIKV transmission and to determine if CHIKV-induced apoptosis or other antiviral mechanisms are responsible. Regardless of the mechanism, our results highlight the need to report transmission of infectious virus and not just viral RNA for laboratory vector competence studies.

Similar rates of infection and transmission of CHIKV RNA have been reported in *Ae*. *aegypti* from southern Mexico orally exposed to IOL [25] and Asian lineage CHIKV [49]. Furthermore, we previously reported that this same mosquito colony from Los Angeles was also highly competent at transmitting Zika virus (ZIKV) in laboratory settings [75]. From 2015–2018, there were 137 and 154 cases of travel-associated CHIKV and ZIKV cases, respectively, reported in Los Angeles county alone [76–78]. Given that CHIKV and ZIKV infections can be asymptomatic [3,79], and therefore are unlikely to be reported, the number of travel-associated infections over that period was probably much greater. Taken together, the evidence of high laboratory vector competence for CHIKV and previously presented for ZIKV, endemicity of *Ae*. *aegypti*, and continued importation of infected travelers support a high risk of local CHIKV and ZIKV transmission in Los Angeles and other cities in southern California.

Oral exposure of mosquitoes to CHIKV fidelity variants that produce more diverse intra-vector populations than WT revealed an association between elevated diversity and attenuated infectivity in laboratory experiments. Focusing on individual mosquito infections, we found that a greater proportion of WT CHIKV infections resulted in low-grade infections (<8 log_10_ genome copies) compared to the P4 and P2P4 fidelity variants. Since this effect was observed in mosquito bodies, we conclude that P4 and P2P4 face a higher barrier to midgut infection than WT, necessitating more robust viral replication in the midgut epithelium to establish infection. Importantly, the lack of shared SNVs or mutational hot-spots in WT CHIKV suggests its more robust infectivity is unlikely to be the result of specific adaptive mutations arising *de novo*. The attenuation of P4 and P2P4 infectivity may be explained by the virus fitness landscape within the midgut epithelium. If the IOL backbone sequence used in this study is maximally fit in terms of midgut infectivity, the potential adaptive benefits of greater diversity are negligible, and the cost of deleterious mutations will be large. The robust infectivity and absence of shared SNVs or mutational hot-spots in WT populations across bodies suggests that the consensus sequence in the bloodmeals used to initiate mosquito infections is indeed highly fit. Future studies of the nsP4 and nsP2 mutations in a less fit consensus sequence would help elucidate whether increased population diversity is detrimental in all CHIKV backbone consensus sequences or dependent upon the initial fitness of the entire population. An alternative explanation for the higher midgut infection barrier in CHIKV P4 and P2P4 compared to WT is that those variants boost antiviral RNA interference (RNAi) responses in midgut epithelial cells. The vital role for RNAi in restricting CHIKV replication has been demonstrated in *Ae*. *aegypti* [80], but not in the midgut epithelium specifically. In further support of this explanation, a fidelity variant of Sindbis virus, another alphavirus, bearing a cysteine-to-glycine substitution at the position analogous to nsP4 C483Y was shown to generate more defective genomes [81], that can be used to generate short-interfering RNAs via a dicer-2-dependent mechanism [82]. Additional studies are warranted to assess whether antiviral RNAi responses are increased in P4 and P2P4-infected midguts compared to WT CHIKV.

The relatively low diversity of WT populations compared to P4 and P2P4 in mosquito bodies did not associate with restricted diversity in expectorated virus. For the mosquito-borne viruses WNV and DENV, high diversity of virus populations in mosquito saliva has been attributed to genetic drift resulting from population bottlenecking and the absence of purifying selection following virus egress [40,42,83,84]. Our observation of more abundant high-frequency SNVs in expectorants compared to bodies indicates these same processes likely contribute to the elevated diversity of CHIKV transmitted in saliva. Whether elevated CHIKV diversity in saliva exerts effects on virus replication following infection of vertebrate hosts remains unclear.

By deep sequencing WT virus populations from bodies at multiple timepoints, we characterized temporal evolution of CHIKV evolution in mosquitoes. We observed a general trend of purifying selection in bodies between 5 and 12 dpf manifesting as a reduction in population diversity. Our results corroborate a recent report with Venezuelan equine encephalitis virus in which a similar decrease in polymorphic sites was observed from 8 to 12 dpf in *Culex taeniopus* mosquitoes [39]. In line with the trend of purifying selection, our analyses of the distribution of polymorphism abundance did not identify any mutational hot-spot regions, but instead identified a number of cold-spots in nsP2, nsP4, capsid, and the three non-coding regions. Of the cold-spots within genes, only the cold-spot at the 3’ end of capsid gene, which encodes the serine protease required for *cis*-autoproteolytic cleavage, has been previously shown to be conserved [85,86].

Across the different CHIKV lineages, 3’ UTRs display substantial phylogenetic diversity due primarily to deletion and/or duplication of large direct repeat sequences (DR) causing variability in 3’ UTR length and organization [38,87]. The frequency of DRs in the 3’ UTR is important for infection of mosquitoes, where deletion of DRs reduces dissemination rates in competitive infection assays [38]. Isolates of ECSA and Asian lineage CHIKV from epidemics in the Americas possess conserved DR sequences [87]. These studies indicate there is functional benefit for CHIKV in duplicating 3’ UTR DRs and conserving their sequences during mosquito infection. Here, we provide further evidence of the importance of CHIKV 3’ UTR sequences by demonstrating relatively high degrees of sequence conservation in the non-coding regions at the subconsensus level within mosquitoes. Similar findings have been reported in cell culture, where serial passage of CHIKV in mosquito and vertebrate cells resulted in the accumulation of few mutations in the 5’ and 3’ UTRs [88]. A caveat of our short-read amplicon sequencing and reference-based read mapping approach is that our analyses of the non-coding regions are unable to capture deletions or insertions of DRs that may have arisen.

In summary, this study is the first to report that: 1) *Ae*. *aegypti* originating from Los Angeles, CA are highly competent laboratory vectors for IOL CHIKV, 2) CHIKV populations with enhanced diversity exhibit attenuated infectivity in mosquitoes, and 3) CHIKV populations are subjected to purifying selection in mosquitoes with substantial sequence conservation in non-coding regions. These results will inform public health risk assessments for CHIKV spread in southern California and advance our understanding of the constraints on CHIKV fitness and evolution in mosquitoes.

## Supporting information

S1 Table Shared and consensus SNVs for WT-infected bodies at 5 or 12 days post-feeding (dpf). Letters indicate the corresponding mosquito identifier. Values reported are allele frequencies in each mosquito. White rectangles indicate the SNV was not present. Site = nucleotide position in genome. Syn/Non = synonymous and non-synonymous.
(TIF)

S1 Fig Infection, dissemination, and transmission of WT and fidelity variant CHIKV at 5 (A) or 12 days post-feeding (B) with discordant values, where CHIKV RNA was detected in L/W and/or saliva but not the corresponding body, included. Samples from individual mosquitoes are arranged horizontally. Cells demarcated with ‘x’ indicate a discordant value that was not included in analyses. LOD = limit of detection.
(TIF)

S2 Fig Lack of correlation between CHIKV genome copies per mosquito body (A, C, E) or saliva expectorant (B, D, F) and Shannon entropy, nucleotide diversity, and substitution frequency for *Ae*. *aegypti* infected with WT or fidelity variant P4 or P2P4. Lines represent the fitted linear regression of all data points. Fitted regression line formulas and R^2^ values are provided. Non-zero slopes for the fitted regression lines were tested by F-test, *p*-values are reported. Nt = nucleotides.
(TIF)

S3 Fig CHIKV genome-wide single nucleotide variant (SNV) mapping shows low-level variation in bloodmeals with evidence of U SNV bias in bloodmeals from replicate experiment 1. Bar colors indicate the nucleotide of the SNV allele. X-axis is the nucleotide position on the reference genome with the start and end of genome elements marked by dashes. nsP = non-structural protein, C = capsid, E = envelope, UTR = untranslated region.
(TIF)

S4 Fig CHIKV genome-wide single nucleotide variant (SNV) mapping from individual mosquito bodies at 12 days post-feeding shows greater variance in P4- and P2P4-infected bodies. Each graph is labeled with the corresponding mosquito letter identifier. Bar colors indicate the nucleotide of the SNV allele. X-axis is the nucleotide position on the reference genome with the start and end of genome elements marked by dashes. nsP = non-structural protein, C = capsid, E = envelope, UTR = untranslated region.
(TIF)

S5 Fig CHIKV genome-wide single nucleotide variant (SNV) mapping from individual mosquito saliva at 12 days post-feeding shows high levels of variance across treatment groups. Each graph is labeled with the corresponding mosquito letter identifier. Bar colors indicate the nucleotide of the SNV allele. X-axis is the nucleotide position on the reference genome with the start and end of genome elements marked by dashes. nsP = non-structural protein, C = capsid, E = envelope, UTR = untranslated region.
(TIF)

S6 Fig CHIKV mutational spectra for (A) bloodmeals, and (B) mosquito bodies and (**C**) saliva at 12 days post-feeding. Frequency of specific mutations across all possible sites are reported for the twelve nucleotide substitutions. Mean frequencies were compared to the WT group by Kruskal-Wallis test with Dunn’s multiple comparisons (* *p*<0.05, ** *p*<0.01). Lines and error bars represent mean frequencies and standard deviation. For G>U in (A), individual bloodmeals are labeled with the corresponding replicate experiment.
(TIF)
