# Supplementary figures and images for: Chikungunya virus populations experience diversity- dependent attenuation and purifying intra-vector selection in Californian Aedes aegypti mosquitoes
Source: PLoS Negl Trop Dis. 2019 Nov 21;13(11):e0007853. doi: 10.1371/journal.pntd.0007853 (PMC6894883; doi:10.1371/journal.pntd.0007853)

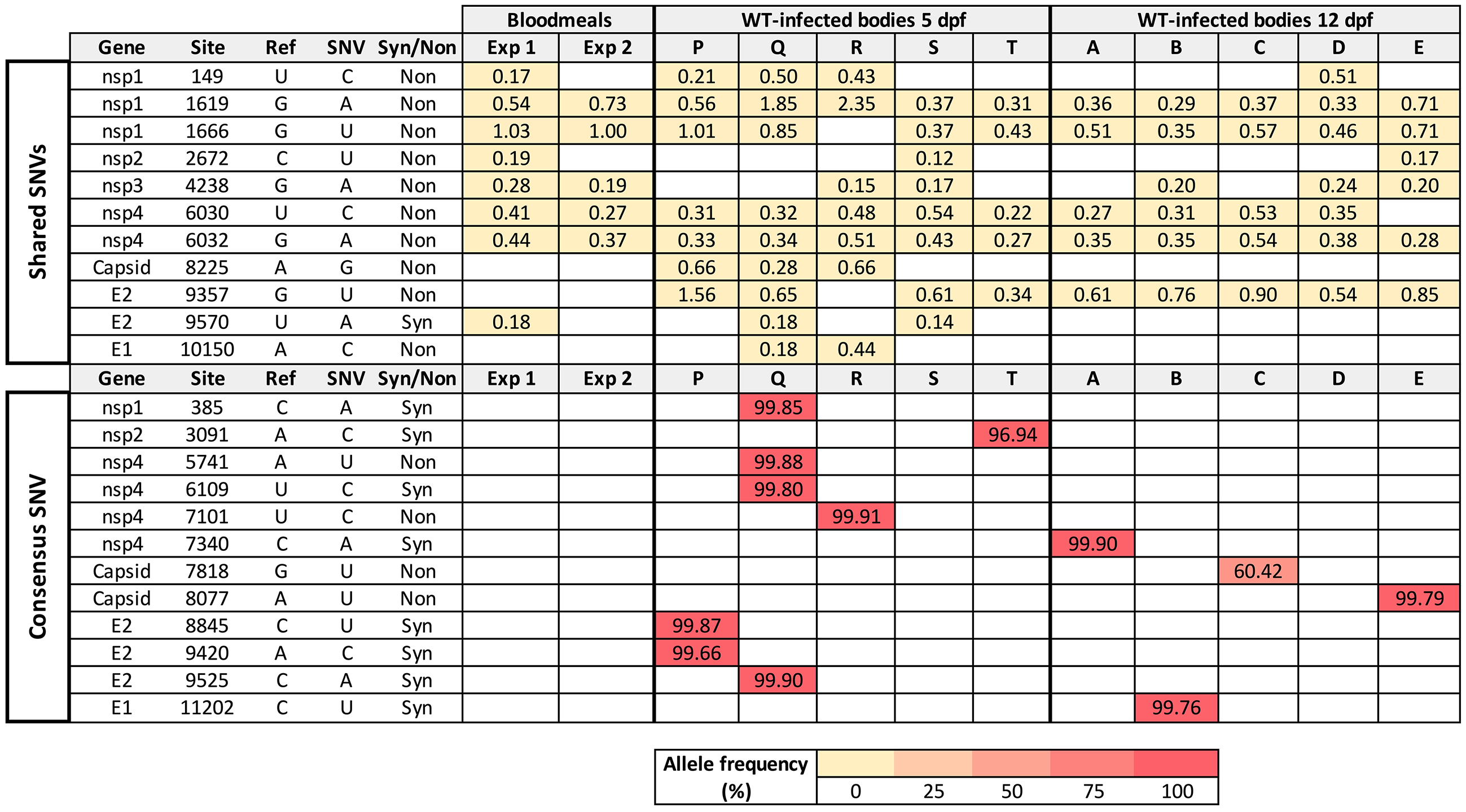

Supplement: S1 Table — Letters indicate the corresponding mosquito identifier. Values reported are allele frequencies in each mosquito. White rectangles indicate the SNV was not present. Site = nucleotide position in genome. Syn/Non = synonymous and non-synonymous. (TIF) [file pntd.0007853.s001.tif]

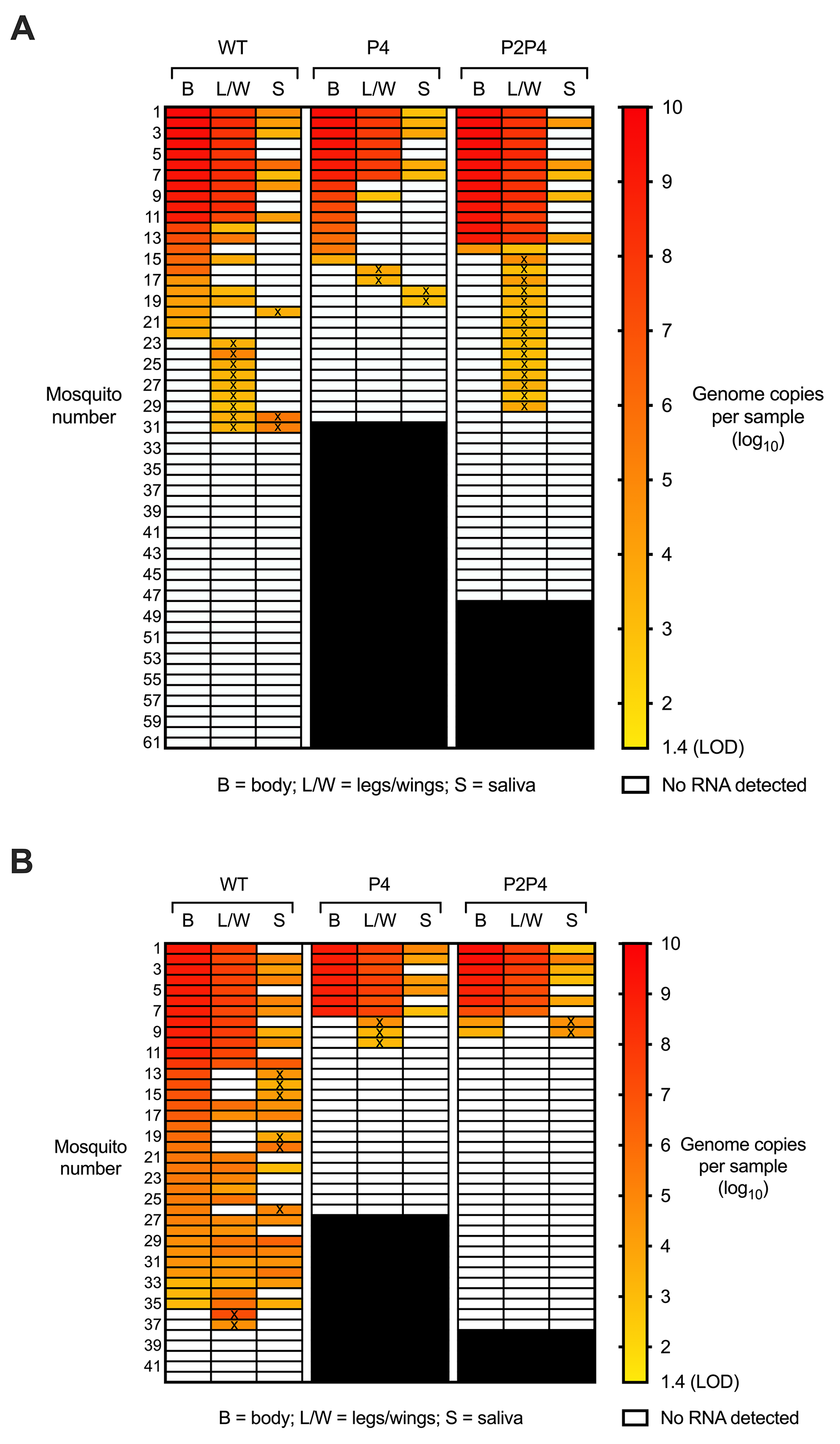

Supplement: S1 Fig — Infection, dissemination, and transmission of WT and fidelity variant CHIKV at 5 (A) or 12 days post-feeding (B) with discordant values, where CHIKV RNA was detected in L/W and/or saliva but not the corresponding body, included. Samples from individual mosquitoes are arranged horizontally. Cells demarcated with ‘x’ indicate a discordant value that was not included in analyses. LOD = limit of detection. (TIF) [file pntd.0007853.s002.tif]

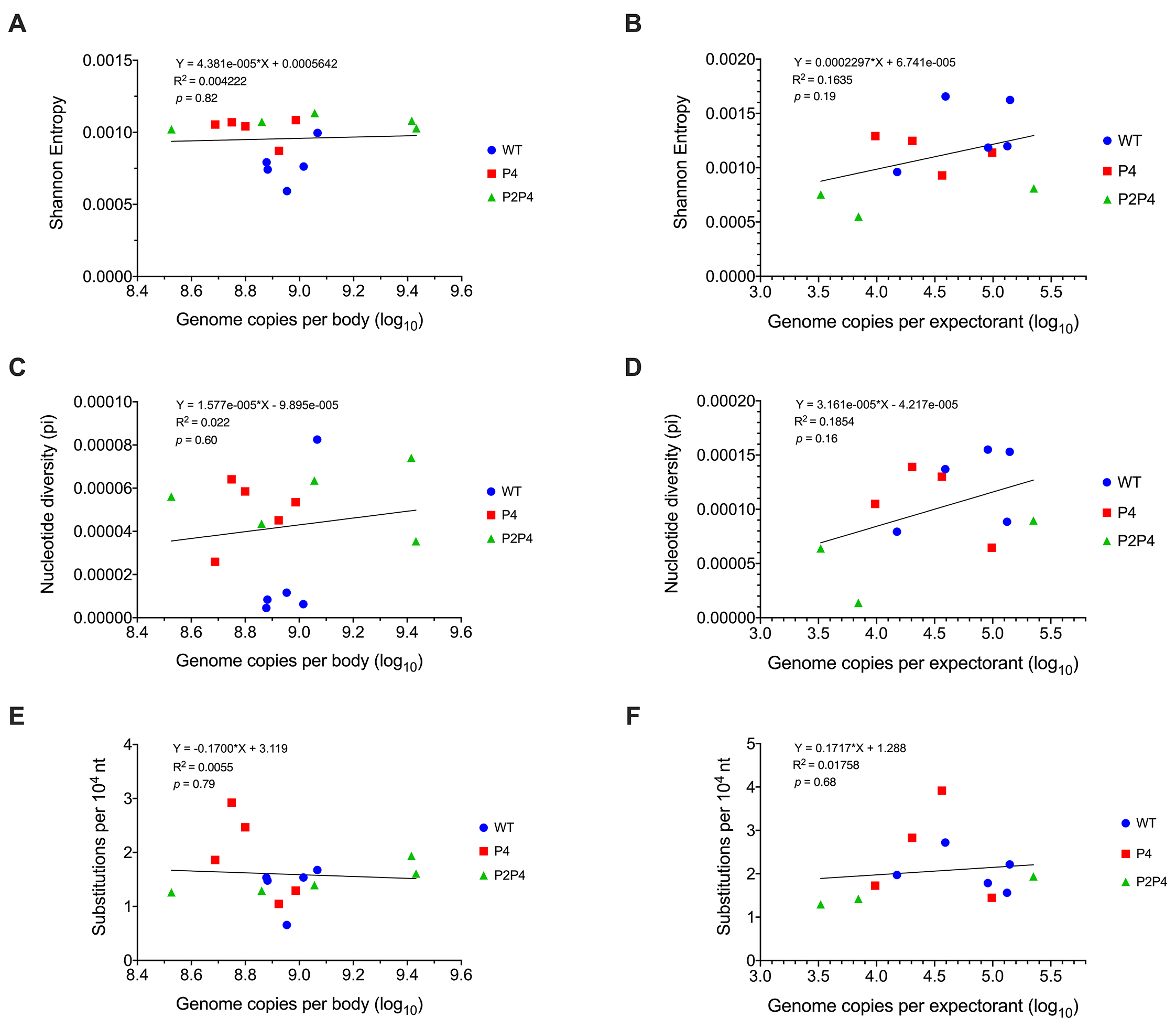

Supplement: S2 Fig — Lack of correlation between CHIKV genome copies per mosquito body (A, C, E) or saliva expectorant (B, D, F) and Shannon entropy, nucleotide diversity, and substitution frequency for Ae. aegypti infected with WT or fidelity variant P4 or P2P4. Lines represent the fitted linear regression of all data points. Fitted regression line formulas and R2 values are provided. Non-zero slopes for the fitted regression lines were tested by F-test, p-values are reported. Nt = nucleotides. (TIF) [file pntd.0007853.s003.tif]

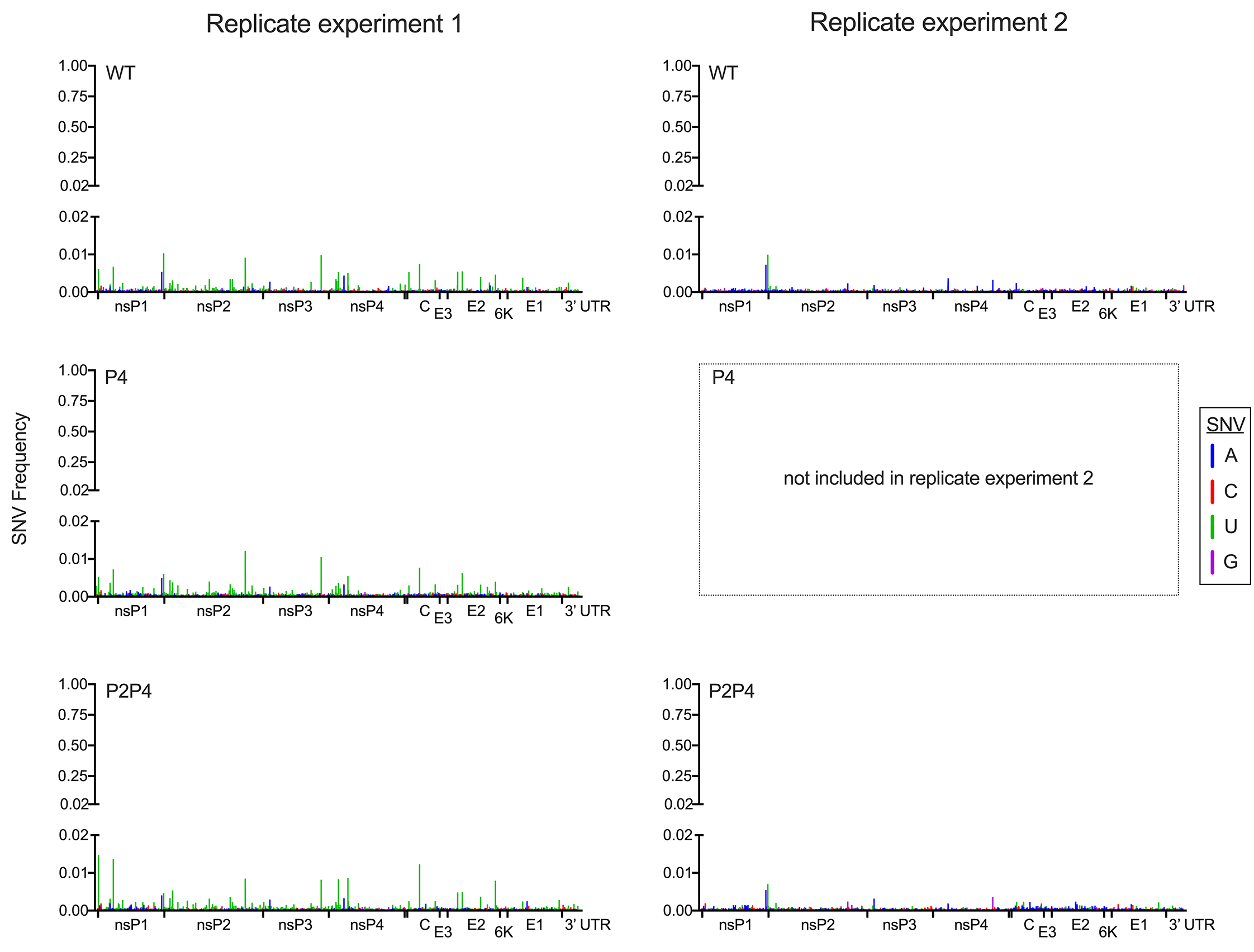

Supplement: S3 Fig — Bar colors indicate the nucleotide of the SNV allele. X-axis is the nucleotide position on the reference genome with the start and end of genome elements marked by dashes. nsP = non-structural protein, C = capsid, E = envelope, UTR = untranslated region. (TIF) [file pntd.0007853.s004.tif]

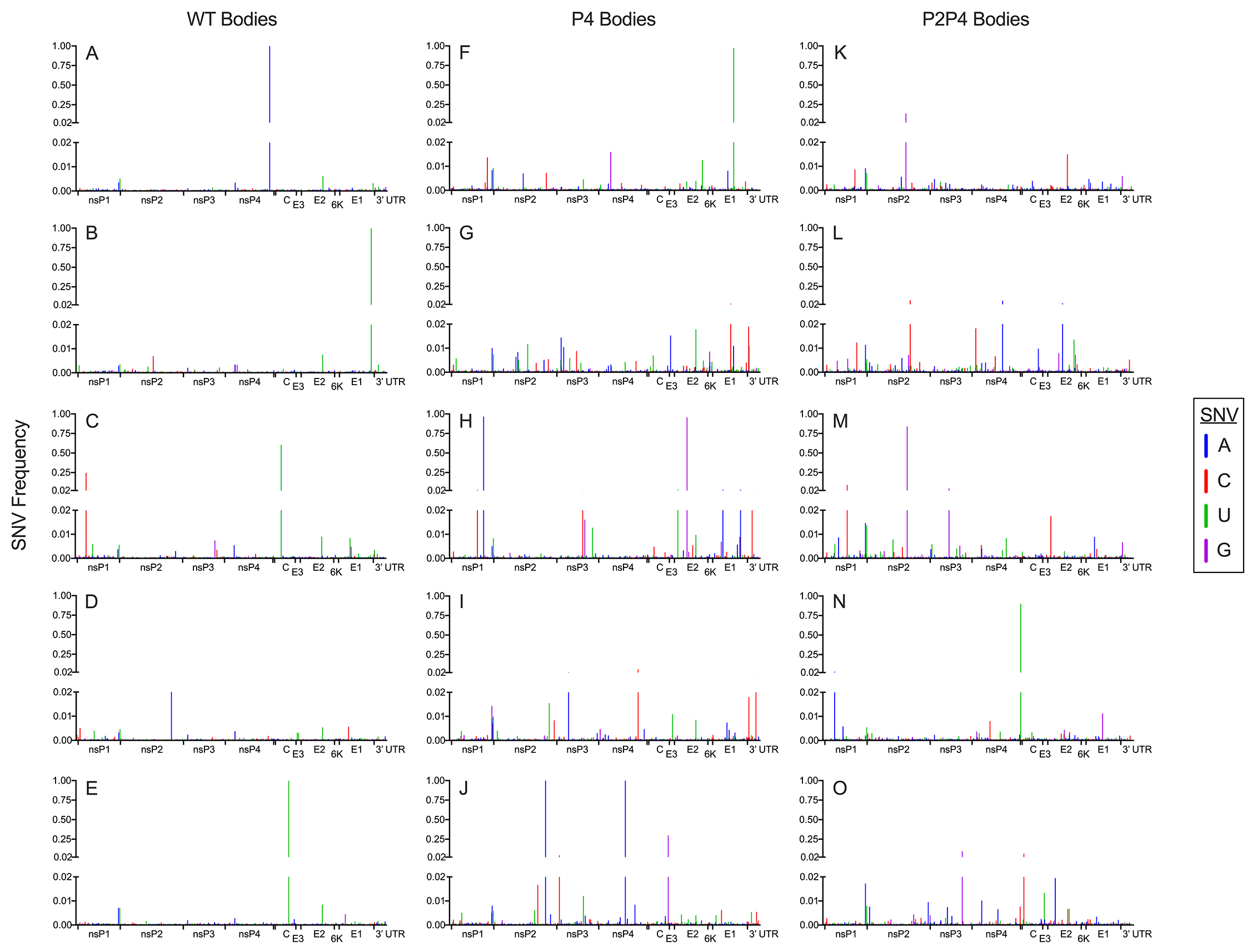

Supplement: S4 Fig — Each graph is labeled with the corresponding mosquito letter identifier. Bar colors indicate the nucleotide of the SNV allele. X-axis is the nucleotide position on the reference genome with the start and end of genome elements marked by dashes. nsP = non-structural protein, C = capsid, E = envelope, UTR = untranslated region. (TIF) [file pntd.0007853.s005.tif]

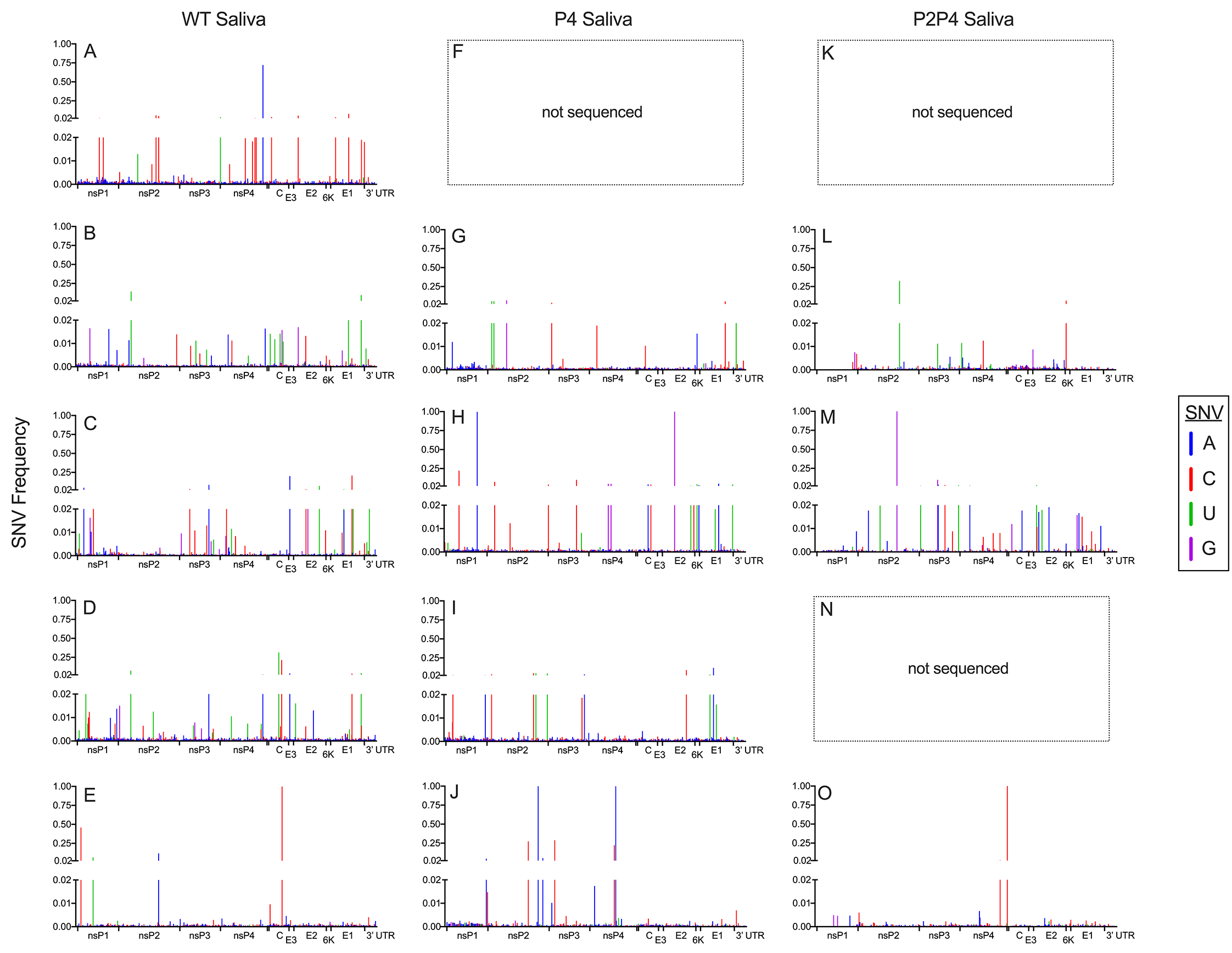

Supplement: S5 Fig — Each graph is labeled with the corresponding mosquito letter identifier. Bar colors indicate the nucleotide of the SNV allele. X-axis is the nucleotide position on the reference genome with the start and end of genome elements marked by dashes. nsP = non-structural protein, C = capsid, E = envelope, UTR = untranslated region. (TIF) [file pntd.0007853.s006.tif]

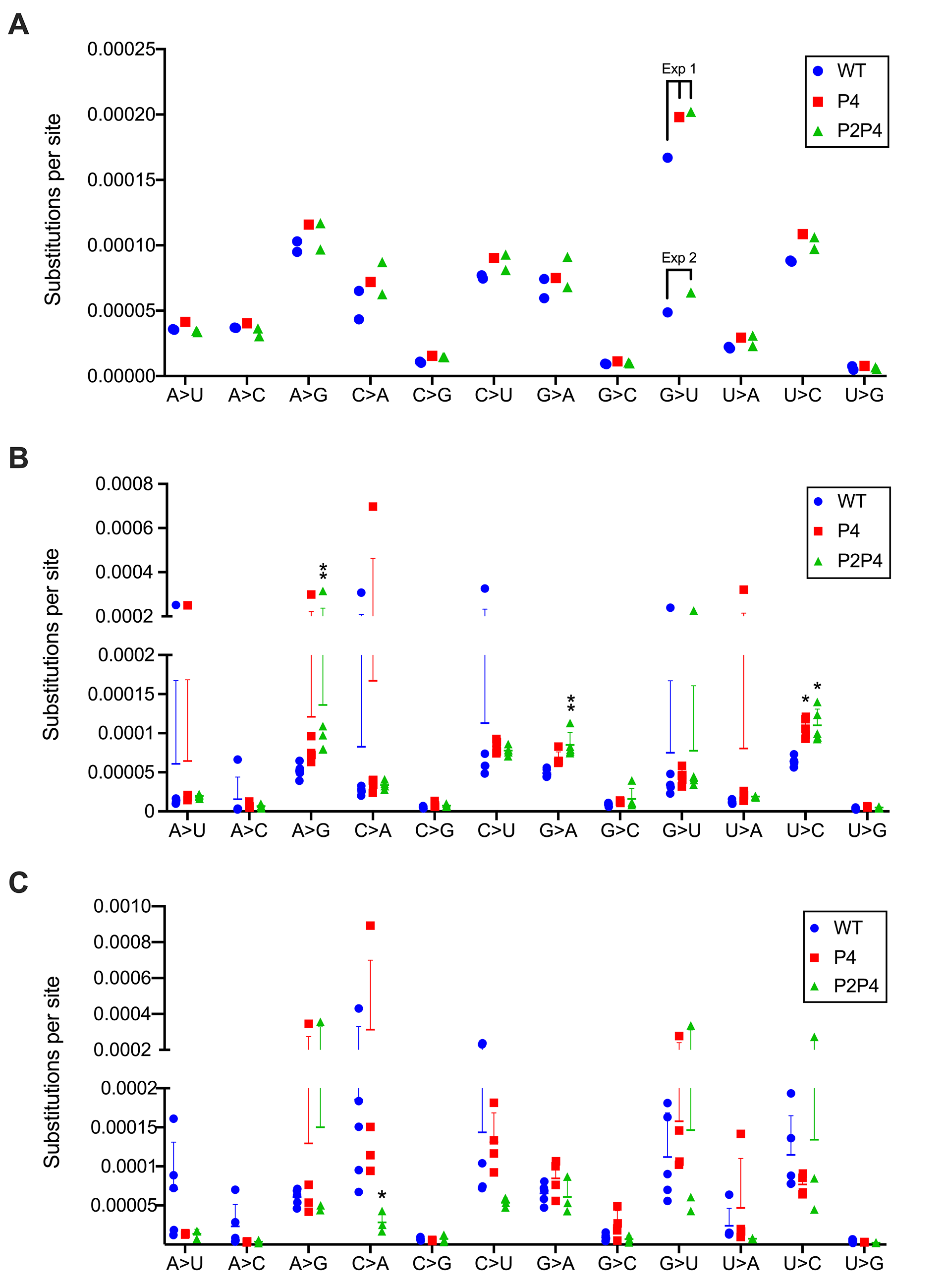

Supplement: S6 Fig — CHIKV mutational spectra for (A) bloodmeals, and (B) mosquito bodies and (C) saliva at 12 days post-feeding. Frequency of specific mutations across all possible sites are reported for the twelve nucleotide substitutions. Mean frequencies were compared to the WT group by Kruskal-Wallis test with Dunn’s multiple comparisons (* p<0.05, ** p<0.01). Lines and error bars represent mean frequencies and standard deviation. For G>U in (A), individual bloodmeals are labeled with the corresponding replicate experiment. (TIF) [file pntd.0007853.s007.tif]
